# N-acetylcysteine downregulates phosphorylated p-38 expression but does not reverse the increased superoxide anion levels in the spinal cord of rats with neuropathic pain

**DOI:** 10.1590/1414-431X20165801

**Published:** 2017-02-16

**Authors:** A. Horst, J.A. de Souza, M.C.Q. Santos, A.P.K. Riffel, C. Kolberg, M.F.M. Ribeiro, L.S. de Fraga, W.A. Partata

**Affiliations:** 1 Laboratório de Neurobiologia Comparada, Departamento de Fisiologia, Instituto de Ciências Básicas da Saúde, Universidade Federal do Rio Grande do Sul, Porto Alegre, RS, Brasil; 2 UNIVATES, Lajeado, RS, Brasil

**Keywords:** Sciatic functional index, Chronic constriction, Sciatic nerve, Mitogen-activated protein kinase, Antinociception, Mechanical threshold

## Abstract

We determined the effect of N-acetylcysteine (NAC) on the expression of the phosphorylated p38 (p-p38) protein and superoxide anion generation (SAG), two important players in the processing of neuropathic pain, in the lumbosacral spinal cord of rats with chronic constriction injury (CCI)-induced neuropathic pain. The sciatic functional index (SFI) was also measured to assess the functional recovery post-nerve lesion. Thirty-six male Wistar rats were divided equally into the following groups: Naive (rats did not undergo surgical manipulation); Sham (rats in which all surgical procedures involved in CCI were used except the ligature), and CCI (rats in which four ligatures were tied loosely around the right common sciatic nerve), which received 2, 4, or 8 intraperitoneal injections of NAC (150 mg·kg^-1^·day^-1^) or saline beginning 4 h after CCI. Rats were sacrificed 1, 3, and 7 days after CCI. The SFI was measured on these days and the lumbosacral spinal cord was used for analysis of p-p38 expression and SAG. CCI induced a decrease in SFI as well as an increase in p-p38 expression and SAG in the spinal cord. The SFI showed a partial recovery at day 7 in saline-treated CCI rats, but recovery was improved in NAC-treated CCI rats. NAC induced a downregulation in p-p38 expression at all time-points evaluated, but did not reverse the increased SAG induced by CCI. Since p-p38 is a mediator in neuropathic pain and/or nerve regeneration, modulation of this protein may play a role in NAC-induced effects in CCI rats.

## Introduction

Accumulating evidence suggests that the protein p38, a member of the family of mitogen-activated protein kinases (MAPK), contributes to neuropathic pain processing, as do nitric oxide (NO) and reactive oxygen species such as superoxide radicals (1–4). N-acetylcysteine (NAC) is a sulfhydryl donor antioxidant that contributes to the regeneration of glutathione and plays a protective role in neurons of the nervous system (5). NAC also induces antinociception in rats with chronic constriction injury (CCI) of the sciatic nerve (6). CCI, one of the most commonly employed animal models of neuropathic pain, simulates the symptoms of chronic nerve compression that correspond to causalgia or complex regional pain syndrome in patients (7).

Recently, it was demonstrated that NAC treatment reduced NO metabolites in the lumbosacral spinal cord of CCI rats (6). These authors suggested that the inhibition of the p38 protein might have contributed to this reduction in NO metabolites. Since MAPKs are activated by upstream kinases via phosphorylation (8), our study assessed the effect of intraperitoneal administration of NAC (150 mg·kg^-1^·day^-1^), given for 1, 3, or 7 days, on the expression of the phosphorylated p38 (p-p38) protein in the lumbosacral spinal cord of rats with CCI. Since NAC reacts relatively slowly with superoxide (5), and we were unable to find a report on the effect of NAC treatment on superoxide anion generation (SAG) in the spinal cord of rats with CCI, our study also evaluated the effect of the administration of NAC on SAG, using the same periods of treatment as above. We also assessed the effects of systemic administration of NAC, given for 1, 3, and 7 days at the same dose, on the sciatic functional index (SFI) in CCI rats, as a proof of functional recovery post-nerve lesion. Analysis of the free walking pattern is a commonly used tool to assess the function of innervated target organs after nerve injury (9).

## Material and Methods

### Animals

All animal procedures were approved by the Animal Ethics Committee of the Universidade Federal do Rio Grande do Sul, Brazil (#23407). Thirty-six adult male Wistar rats, weighing 200-300 g, were divided into three experimental groups (Naive, Sham and CCI), and each one was further divided into two subgroups (n=6 in each subgroup), which received NAC (Fluimucil¯, Zambon Laboratórios Farmacêuticos Ltda., Brazil) at a dose of 150 mg·kg^-1^·day^-1^ (6,10,11) or 0.9% saline solution. Rats received 2, 4, or 8 intraperitoneal injections (one injection for day) of saline or NAC beginning 4 h after CCI. Thus, rats were sacrificed 1, 3, and 7 days after CCI (6). Rats were not anesthetized for the injections.

### Induction of chronic constriction injury (CCI) and mechanical threshold assessment

After anesthesia (90 mg/kg ketamine and 10 mg/kg xylazine), the right common sciatic nerve was exposed proximal to its trifurcation, and four ligatures (4.0 Shalon chromic catgut, Brazil) were tied loosely around it as described by Bennett and Xie (12), with slight modifications according to Horst et al. (6). To expose the sciatic nerve of Sham rats, all surgical procedures involved in CCI were used except the ligature.

Rats were subjected to sensitivity assessments before the surgical procedure (day 0) and 1, 3, and 7 days after surgery as described by Horst et al. (6). To measure mechanical sensitivity, responses of the injured hind paw to a range of applied innocuous von Frey filaments (North Coast Medical, Inc., USA) were evaluated. The minimum and maximum stimulus intensity was 1 and 64 g, respectively. The first stimulus was always initiated with the lowest filament. If there was no positive response, the next higher filament was applied. This testing pattern was continued until a withdrawal response was recorded.

### Sciatic functional index (SFI)

Recovery of the right hind limb locomotor activity was monitored by analysis of the free walking pattern according to de Medinaceli et al. (13). The rats’ footprints were used to determine the following measurements: 1) distance from the heel to the third toe (the print length, PL); 2) distance from the first to the fifth toe (the toe spread, TS), and 3) distance from the second to the fourth toe (the intermediate toe spread, ITS). These 3 measurements were obtained from the experimental (E) and normal (N) sides. Several prints of each foot were obtained on each track, but only 3 prints of each foot were used to determine the mean of measurements on the experimental and normal sides. These means were included in the formula for the sciatic functional index (SFI): SFI = -38.3 (EPL - NPL) / NPL + 109.5(ETS – NTS) / NTS + 13.3(EIT – NIT) / NIT - 8.8

The result was considered to be an index of the functional condition of the sciatic nerve, where zero (±11) represented the normal function and about -100 represented the loss of function resulting from CCI. The SFI was assessed in all groups at days 1, 3, and 7. These tests were conducted at the same time of day (7:00 am) by the same researcher.

### Western blotting

To identify the expression of p-p38 and glyceraldehyde-3-phosphate dehydrogenase (GAPDH), rats were sacrificed by decapitation and the lumbosacral spinal cord was promptly removed, immediately frozen in liquid nitrogen, stored at -80° C prior to processing, and homogenized in lysis buffer. Forty-five μg of protein was separated on a 10% SDS-PAGE gel and blotted onto a nitrocellulose membrane. The membranes were blocked with 10% nonfat dry milk and then incubated overnight at 4°C with rabbit anti-p-p38 (1:100; Merck Millipore, USA) and mouse anti-GAPDH (1:2000; Merck Millipore). Immunoreactive bands were detected using secondary antibodies and ECL (Merck Millipore), followed by exposing the membrane to autoradiography film (GE Healthcare Limited, UK).

### Estimation of superoxide anion generation (SAG)

The lumbosacral spinal cord SAG was estimated using the reduced nitroblue tetrazolium (NBT) method of Wang et al. (14). Briefly, sections of fresh tissue from the lumbosacral spinal cord reacted with NBT to form formazan as an index of superoxide anion generation. The absorbance of formazan was determined spectrophotometrically at 540 nm.

The quantity of NBT reduction = A × V / (T × Wt × ε × l), where A is the absorbance of blue formazan at 540 nm, V is the volume of the solution, T is the time period (90 min) during which the rings were incubated with NBT, Wt is the blotted wet weight of the spinal cord portion, ε is the extinction coefficient of blue formazan (i.e., 0.72 L·mmol^-1^·mm^-1^), and l is the length of the light path. Results are reported as picomoles per minute per milligram of wet tissue.

### Statistical analysis

The results were analyzed using three-way ANOVA (factors: lesion, treatment and time) followed by the Tukey *post hoc* test. Differences were considered to be statistically significant when P<0.05.

## Results

**Figure 1:**
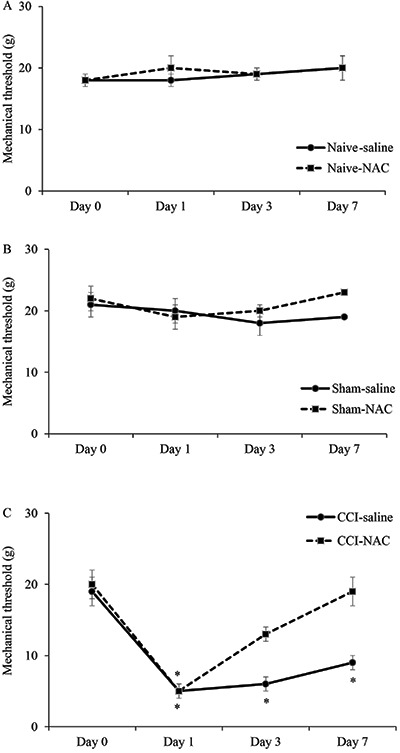
Assessment of mechanical threshold in rats with chronic constriction injury (CCI) of the sciatic nerve that received 2, 4, or 8 intraperitoneal injections of saline or N-acetylcysteine (NAC; 150 mg·kg^-1^·day^-1^) beginning 4 h after CCI. Rats were sacrificed 1, 3, and 7 days after surgery. In Sham rats, all surgical procedures involved in the CCI were used except the ligature. Day 0: sensitivity assessments before the surgical procedure. Data are reported as means±SE. *P<0.05 compared to pre-nerve lesion values (repeated-measures ANOVA followed by Tukey *post hoc* test, n=6).

The mechanical threshold did not change significantly in the Naive and Sham groups (Figure 1A and B). After CCI, rats exhibited increases in the mechanical sensitivity (Figure 1C). At days 1 and 3, the mechanical threshold of the saline-treated CCI rats decreased 80% compared to pre-nerve lesion levels. Similar responses were found at day 7. While the mechanical threshold of the NAC-treated CCI rats decreased 80% at day 1, an antinociceptive effect was apparent 3 days after the NAC administration. At this time-point the reduction was 30% in the mechanical threshold of the NAC-treated rats, compared to pre-nerve lesion levels. Compared to saline-treated CCI rats, NAC-treated rats showed an improvement of 133% in the mechanical threshold at day 3. At 7 days after CCI, the mechanical threshold was similar to the pre-nerve lesion level.

**Figure 2:**
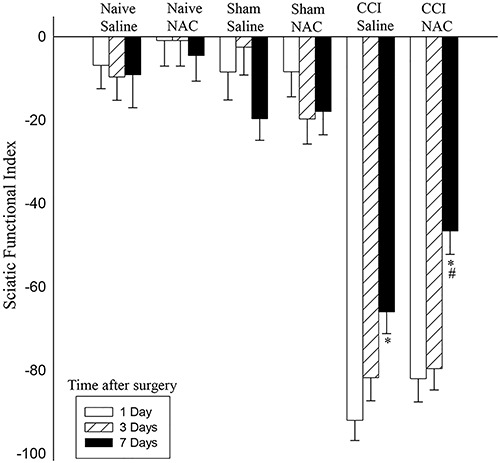
Functional recovery assessed by the Sciatic Functional Index (SFI) in rats with chronic constriction injury (CCI) of the sciatic nerve that received 2, 4, or 8 intraperitoneal injections of saline or N-acetylcysteine (NAC; 150 mg·kg^-1^·day^-1^) beginning 4 h after CCI. Rats were sacrificed 1, 3, and 7 days after surgery. On the Y axis, the results are reported in units, and 0 indicates normality and -100 the total absence of functionality. Data are reported as means±SE. *P<0.05 compared to CCI group at day 1; ^#^P<0.05 compared to saline-treated CCI group at day 7 (three-way ANOVA followed by Tukey *post hoc* test).

**Figure 3:**
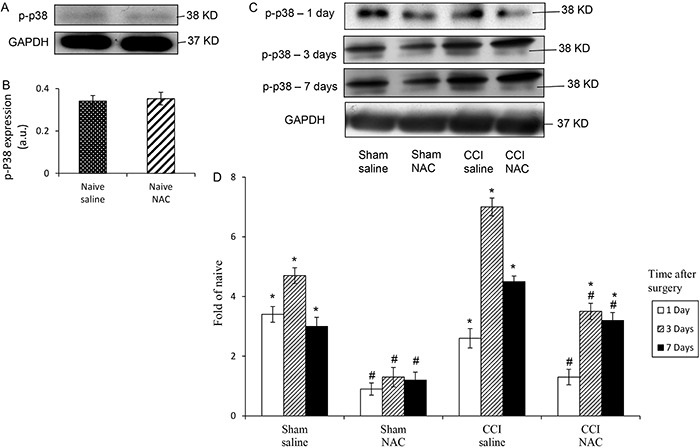
Phosphorylated p-38 (p-p38) expression in the spinal cord of rats with chronic constriction injury (CCI) of the sciatic nerve that received 2, 4 or 8 intraperitoneal injections of saline (vehicle) or N-acetylcysteine (NAC; 150 mg·kg^-1^·day^-1^) beginning 4 h after CCI. Rats were sacrificed at 1, 3, and 7 days after surgery. Western blot bands (*A*, *C*) and a data summary (*B*, *D*) are shown. GAPDH served as loading control. Data are reported as means±SE in arbitrary units (a.u.) or fold of Naive. *P<0.05 compared to the Naive group at the same time-point. ^#^P<0.05 compared to the group that received only saline at the same time-point (three-way ANOVA followed by Tukey *post hoc* test).

SFI values for Sham groups were near zero at all time-points, indicating normal sciatic nerve function. One day after surgery, the walking pattern of all CCI rats showed a dramatic decrease in SFI (Figure 2). The values were near -100, indicating complete loss of sciatic nerve function. At this time-point, the SFI values showed no significant difference among CCI rats. At postoperative day 3, the SFI values were still decreased and no significant change was found in the CCI rats. However, 7 days after the injury, SFI values showed some recovery. While the recovery was 28% in saline-treated CCI rats, the recovery was 44% in NAC-treated CCI rats, compared to values from CCI rats at day 1. Comparison of saline-treated CCI rats with NAC-treated CCI rats at day 7 showed that SFI increased approximately 30% in CCI rats that received the NAC treatment, indicating that NAC improved the recovery of the SFI in rats with an injured sciatic nerve.

p-p38 expression did not change significantly in any of the Naive rats. After sham surgery, saline-treated rats exhibited increases in p-p38 expression at days 1, 3, and 7 (Figure 3) compared to Naive rats. After NAC treatment, no significant difference was found between Sham and Naive rats in p-p38 expression at days 1, 3, and 7. Compared to saline, NAC-treated Sham rats showed a reduction of around 65% in p-p38 expression.

After CCI, rats exhibited increases of 150, 60, and 350% in p-p38 expression at days 1, 3, and 7, respectively, compared to Naive rats. NAC treatment reduced the expression of p-p38 by 46, 50, and 30% at days 1, 3, and 7, respectively, compared to saline-treated CCI rats. Comparing Naive rats and NAC-treated CCI rats, the treatment induced a reduction of around 187% in p-p38 expression at days 3 and 7. At day 1, no significant difference was found between NAC-treated CCI rats and Naive rats.

**Figure 4:**
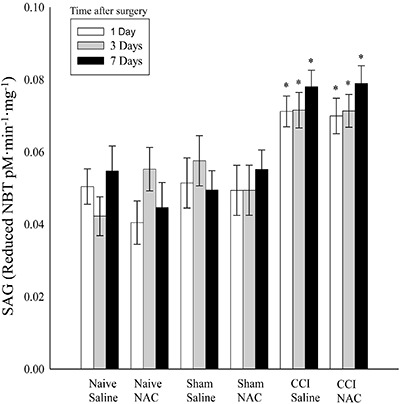
Superoxide anion generation (SAG) in the spinal cord of rats with chronic constriction injury (CCI) of the sciatic nerve that received 2, 4 or 8 intraperitoneal injections of saline or N-acetylcysteine (NAC; 150 mg·kg^-1^·day^-1^) beginning 4 h after CCI. Rats were sacrificed 1, 3, and 7 days after surgery. In Sham rats, all surgical procedures involved in the CCI were used except the ligature. Data are reported as means±SE. *P<0.05 compared to the Naive and Sham groups (three-way ANOVA followed by Tukey *post hoc* test).

The level of SAG increased significantly in the lumbosacral spinal cord of the CCI rats (Figure 4). The SAG increased around 40% in the spinal cord of saline-treated CCI rats at days 1 and 3, respectively, and 53% at day 7. Similar percentages were found in the spinal cord of NAC-treated CCI rats, showing that this treatment did not change SAG in the spinal cord. No significant change was found in the Sham groups.

## Discussion

The antinociceptive effect of the intraperitoneal administration of NAC is in line with a previous study that used rats submitted to CCI (6). Thus, our results support the antinociceptive effect of NAC.

Our results showed that on day 7 after surgery, SFI was improved in the CCI rats. Moreover, the NAC-treated CCI rats showed a much better recovery. The SFI is a reliable, repeatable, economical, and quantitative method of evaluating function following sciatic nerve injury (9). Since CCI-induced foot deformities appear to be more related to spontaneous ongoing pain (15), our results suggest that the improved SFI in the NAC-treated CCI rats may be related to attenuation of the spontaneous ongoing pain. In fact, the NAC treatment attenuated the reduction in the mechanical threshold induced by CCI 3 days after the beginning of NAC treatment, but this threshold was similar to pre-nerve lesion level at day 7 (6).

Previous studies have shown that activation of p38 MAPK is involved in neuropathic pain (16,17). A previous study found a significant increase in spinal p-p38 expression at day 3, which was maintained for 2 weeks in rats with CCI (17). Although no information is available, we speculate that this increase might also be present at day 1. In rats with ligation of the L5 spinal nerve, another model of neuropathic pain, p-p38 expression was significantly increased in the spinal cord at day 1 (18). As hypothesized, we detected an increase in p-p38 expression in the spinal cord of the CCI rats at days 1, 3, and 7. This increase might be involved with the critical role of p38 MAPK in regeneration of the sciatic nerve. It has been demonstrated that p38 MAPK has an important physiological role in nerve regeneration and may be important for controlling both initiation of inflammation and recovery from nerve injury (9). This physiological role may contribute to the improved SFI in saline-treated CCI rats.

NAC, in turn, induced downregulation of p-p38 expression at all time-points evaluated. This reduction could play a role in the antinociceptive effect of NAC in rats with CCI described by Horst et al. (6). Administration of a p-38 inhibitor has been shown previously to reduce hyperalgesia after nerve injury (17). Nevertheless, the effect of NAC on p-p38 expression may be related to the neuroprotector role of these molecules. NAC provides a highly significant effect of neuroprotection in animal nerve injury model (19). p38 MAPK plays critical roles in the differentiation and/or survival of neurons (9). It is probable that additive effects of NAC and p-p38 may have contributed to the decrease in p-p38 expression. The antinociceptive and/or neuroprotector effects of NAC may have contributed to the better recovery in SFI on day 7 after CCI.

It is possible that the reduction in p-p38 may contribute to a decrease in NO metabolites in the spinal cord of rats with CCI, as suggested by Horst et al. (6). It has been demonstrated that neuronal NO synthase, one of the enzymes responsible for NO production, is important for spinal microglial activation after nerve injury (20) and microglial p-p38 plays an important role in CCI-induced nociception (1). However, further studies are necessary to clarify the relationship between NAC, p-38 and NO in the spinal cord of CCI rats.

The increase in SAG in the spinal cord of the saline-treated CCI rats may also be related to pain. Significant evidence links the superoxide anion to pain of several etiologies, including neuropathic pain (21). However, NAC did not reduce the increase in SAG in the spinal cord of rats with CCI. This result may be related to the low capacity of NAC to react with superoxide anions (5).

The reduction in the mechanical threshold in the Sham rats may be due to the procedures involving manipulation of deep tissues, such as muscles and adjacent connective tissue, which induce pain (22). Since NAC also induced downregulation of p-p38 expression in these animals, we suggest that this result reinforces the antinociceptive effect of the NAC in pain conditions.

In summary, our study showed that NAC modulated p-p38 expression in the spinal cord of rats with CCI, but it was unable to reverse the increase of SAG levels induced by CCI. Since p-p38 is an important mediator in neuropathic pain and nerve regeneration, the modulation of this protein may contribute to NAC-induced analgesia and/or functional nerve recovery after CCI.
